# Hemophagocytic Lymphohistiocytosis: A Life-Threatening Hyperinflammatory Syndrome

**DOI:** 10.7759/cureus.92820

**Published:** 2025-09-21

**Authors:** Tehreem Khan, Laraib Arshad, Huria Huma, Maham R Sidhu, Leena George

**Affiliations:** 1 Respiratory Medicine, Glenfield Hospital, Leicester, GBR; 2 Pulmonology, Glenfield Hospital, Leicester, GBR

**Keywords:** adult-onset still’s disease, anakinra, critical care, hemophagocytic lymphohistiocytosis (hlh), hyperferritinemia, interstitial lung disease, latent tuberculosis, organizing pneumonia, pancytopenia, parvovirus b19

## Abstract

Hemophagocytic lymphohistiocytosis (HLH) is a rare, life-threatening hyperinflammatory syndrome resulting from uncontrolled immune activation. It can be triggered by infections, malignancies, or autoimmune conditions and is difficult to diagnose due to its nonspecific clinical presentation.

A male patient around 30 years old was admitted to the ICU in 2016 with severe diarrhea, vomiting, and abdominal pain. He developed pancytopenia requiring granulocyte colony-stimulating factor (G-CSF) support. Bone marrow aspiration and trephine revealed hemophagocytosis, with markedly elevated serum ferritin levels and a negative autoimmune workup apart from a positive anti-Ro antibody. The diagnosis of acquired HLH was established in the patient. Latent tuberculosis (TB) was treated empirically, and polymerase chain reaction (PCR) detected parvovirus B19. He was managed with high-dose dexamethasone after a hematology consultation and received intravenous immunoglobulin (IVIG).

A few years later, in 2022, the patient was readmitted with acute respiratory distress and bilateral pulmonary consolidations. He was diagnosed with adult-onset Still’s disease (AOSD) and secondary HLH. Imaging suggested fibrotic organizing pneumonia with a differential diagnosis of interstitial lung disease (ILD) or nonspecific interstitial pneumonitis (NSIP). Both episodes of HLH were successfully managed with interleukin-1 receptor antagonist therapy, i.e., anakinra.

This case demonstrates the complexity of HLH in adults. The first episode was likely to be triggered by the latent TB and parvovirus infection, and the second one by the AOSD. Both episodes were treated with anakinra, showing that targeting specific immune system chemicals can help manage HLH.

Clinicians should consider the possibility of HLH for patients who have low blood counts and widespread inflammation with an unclear cause, especially if they have known risk factors. Early recognition and prompt treatment with medicines that are targeted towards the immune system can save lives. It's also important to monitor these patients over time, as HLH can recur and may lead to long-term problems like lung damage.

## Introduction

Hemophagocytic lymphohistiocytosis (HLH) is a rare, life-threatening hyperinflammatory syndrome caused by excessive immune activation, leading to a cytokine storm and progressive multiorgan dysfunction [1]. HLH is broadly classified as primary (familial) or secondary (acquired). Primary HLH results from inherited defects in cytotoxic function of natural killer (NK) and T cells, typically presenting in infancy or early childhood [1]. In contrast, secondary HLH develops in response to triggers such as infections, malignancies, autoimmune or autoinflammatory diseases, or medications and can occur at any age [1].

The estimated incidence of primary HLH in children is ~1-1.5 per 100,000 annually [1]. In adults, HLH is less well defined, but population studies suggest an incidence of ~0.8-1 per 100,000 per year, with reported mortality of 40-70% depending on the underlying trigger, highest in malignancy-associated HLH [2,3].

The clinical presentation is often nonspecific, including persistent fever, hepatosplenomegaly, cytopenias, hyperferritinemia, and liver dysfunction. Diagnosis is typically guided by the HLH-2004 criteria, which require at least five of eight features, including fever, splenomegaly, cytopenias, hypertriglyceridemia/hypofibrinogenemia, hemophagocytosis, impaired NK cell activity, hyperferritinemia, or elevated soluble interleukin-2 receptor (sIL-2R) [2].

Infections remain the most frequent triggers of secondary HLH, with Epstein-Barr virus (EBV), cytomegalovirus (CMV), and parvovirus B19 commonly implicated [3]. Autoimmune diseases such as systemic lupus erythematosus (SLE) and adult-onset Still’s disease (AOSD) can precipitate a distinctive subtype termed macrophage activation syndrome (MAS). Within rheumatology, MAS is regarded as part of the HLH spectrum but differs from “classic” secondary HLH: it arises in autoimmune or autoinflammatory contexts, tends to be recognized earlier, and is often treated with cytokine-directed biologics rather than cytotoxic chemotherapy [3].

Prompt recognition and treatment are essential. Standard regimens for primary HLH include corticosteroids, etoposide, and cyclosporine, with hematopoietic stem cell transplantation required for a cure. In adults with secondary HLH, particularly MAS, targeted biologic therapies such as anakinra, tocilizumab, and Janus kinase (JAK) inhibitors are increasingly used with encouraging results [4,5]. Emerging evidence supports intravenous anakinra in MAS, demonstrating rapid clinical improvement and a favorable safety profile [6-8].

## Case presentation

A young male in his early 30s was admitted to the ICU in 2016 with a history of profuse diarrhea, persistent vomiting, and severe abdominal pain. On initial evaluation, he was febrile, hypotensive, and appeared acutely unwell. Investigations revealed pancytopenia, with marked leukopenia, anemia, and thrombocytopenia. Inflammatory markers were significantly elevated, with a serum ferritin level exceeding 10,000 µg/L. Liver enzymes were mildly elevated, and coagulopathy was noted. His C-reactive protein (CRP) and lactate dehydrogenase (LDH) levels were also raised.

Based on initial investigations, a bone marrow aspiration and trephine biopsy were performed, which demonstrated hemophagocytosis. Further investigations, including an autoimmune workup, were done. Parvovirus B19 DNA was detected in the blood by polymerase chain reaction (PCR). Latent tuberculosis (TB) was identified through relevant investigations, but there was no evidence of active disease. Autoimmune serology was largely negative, except for positive anti-Ro antibodies.

The patient fulfilled five out of eight HLH-2004 diagnostic criteria, including fever, cytopenias, hyperferritinemia, hemophagocytosis on bone marrow, and elevated triglycerides. A diagnosis of secondary HLH was made. The hematology team was involved, and as per their recommendation, he was treated with intravenous dexamethasone, intravenous immunoglobulin (IVIG), and supportive care, including granulocyte colony-stimulating factor (G-CSF) for neutropenia. Empirical treatment was given for latent TB. The patient responded well to treatment. Table 1 provides a comparison of the two episodes of HLH in the patient.

**Table 1:** Comparison of the two episodes of hemophagocytic lymphohistiocytosis (HLH) in the patient. This table summarizes the clinical presentation, identified triggers, organ involvement, diagnostic findings, imaging results, treatment modalities, and outcomes for two episodes of hemophagocytic lymphohistiocytosis (HLH) in the patient. IVIG: intravenous immunoglobulin; AOSD: adult-onset Still’s disease

Feature	Episode 1 (2016)	Episode 2
Trigger	Hemophagocytic lymphohistiocytosis was triggered by parvovirus B19 and latent tuberculosis (TB) infection.	The episode was triggered by adult-onset Still’s disease (AOSD), an autoinflammatory condition.
Organ involvement	Gastrointestinal tract and bone marrow were involved during this episode.	Lung parenchyma and bone marrow were the primary sites of involvement.
Diagnostic findings	Laboratory investigations showed elevated serum ferritin, cytopenias, and bone marrow hemophagocytosis.	Diagnostic workup revealed hyperferritinemia, cytopenias, and radiological evidence of pulmonary infiltrates.
Imaging	Imaging was not available during this episode.	Computed tomography (CT) imaging revealed organizing pneumonia and fibrotic changes in the lungs.
Treatment	The patient received intravenous dexamethasone and intravenous immunoglobulin (IVIG).	Treatment included anakinra, an interleukin-1 (IL-1) receptor antagonist.

**Figure 1:**
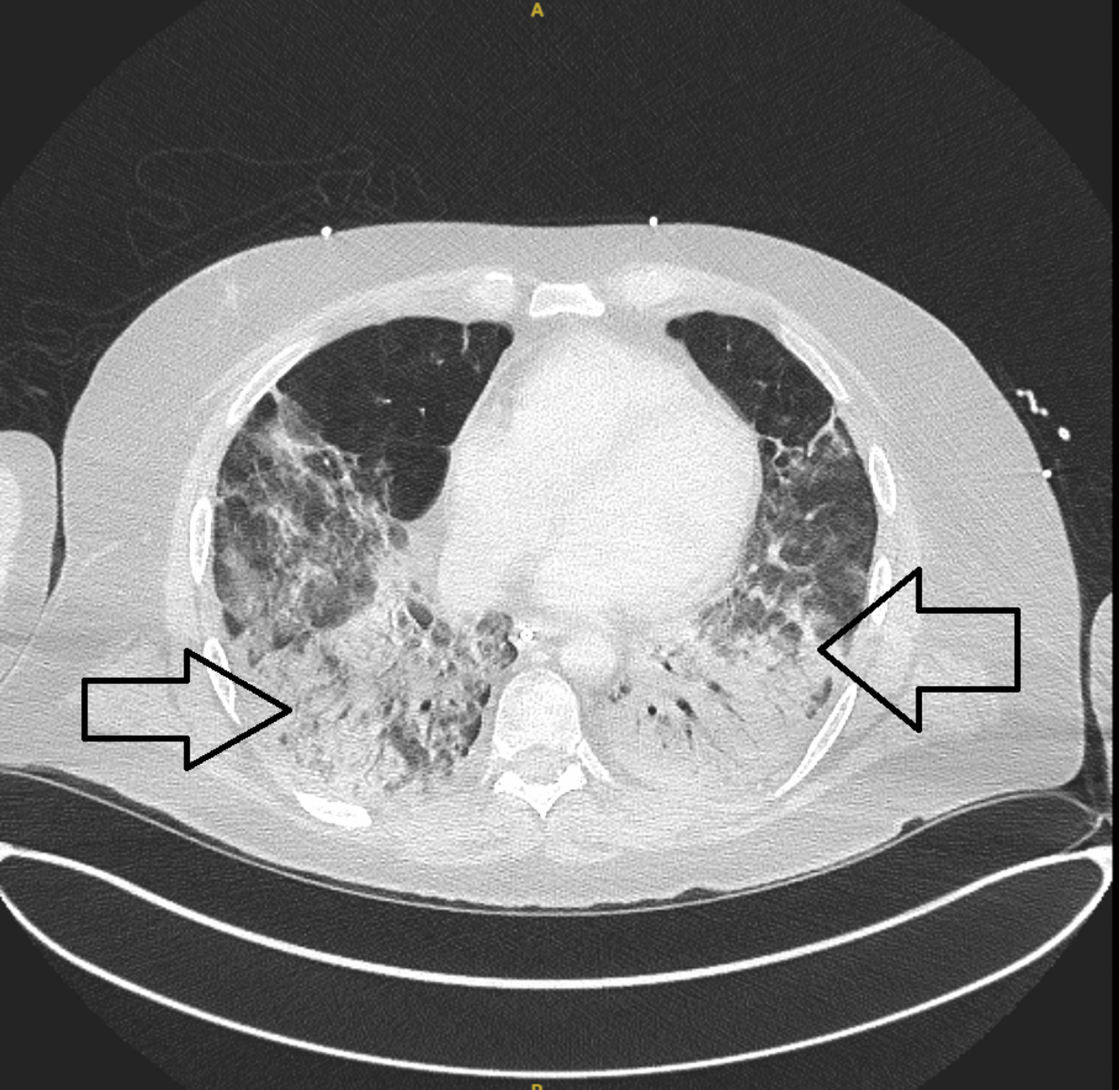
Axial CT chest (August 2022) shows bilateral lower lobe consolidation and ground-glass opacities (black arrows), consistent with organizing pneumonia and active inflammation. This scan corresponds to the initial presentation of hemophagocytic lymphohistiocytosis (HLH), likely infection-triggered. HLH: hemophagocytic lymphohistiocytosis; CT: computed tomography

A few years later, he was readmitted with acute respiratory distress and worsening systemic symptoms. Imaging revealed bilateral pulmonary consolidations and ground-glass opacities, consistent with organizing pneumonia (Figure 1). He was hypoxic and required oxygen therapy. He was referred for extracorporeal membrane oxygenation (ECMO), but didn't require it at the end. Repeated laboratory investigations again showed elevated inflammatory markers and hyperferritinemia. Bone marrow evaluation was not repeated, but the clinical picture was consistent with a recurrence of HLH.

**Figure 2:**
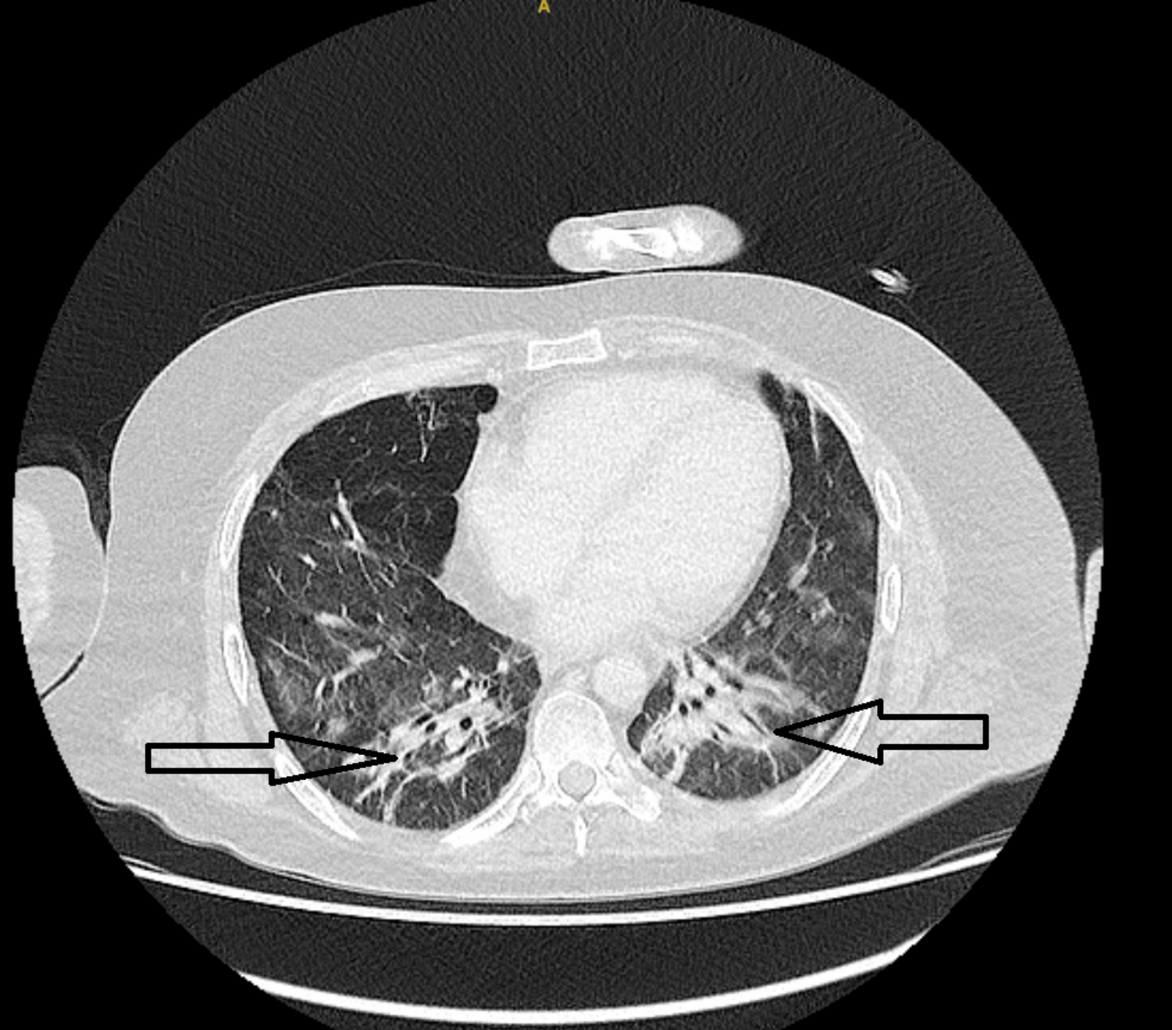
CT chest (Sept 2022, non-contrast) shows interval improvement in pulmonary findings after corticosteroid therapy, with decreased bilateral ground-glass opacities and consolidations. Black arrows indicate residual fibrotic bands and resolving infiltrates, mainly in the lower lobes, reflecting partial resolution of HLH-associated pulmonary changes. NCCT: non-contrast computed tomography

Computed tomography (CT) imaging revealed organizing pneumonia and fibrotic changes in the lungs. Treatment included anakinra, an interleukin-1 (IL-1) receptor antagonist. Figure 2 demonstrates interval improvement in pulmonary infiltrates following corticosteroid therapy.

During this admission, the patient was diagnosed with AOSD, based on high spiking fevers, rash, leukocytosis, and elevated serum ferritin. The differential diagnosis for the pulmonary findings included fibrotic organizing pneumonia, nonspecific interstitial pneumonitis (NSIP), or interstitial lung disease (ILD), possibly related to chronic inflammation or prior HLH episodes.

A diagnosis requires at least five of these criteria. The fulfilment of diagnostic criteria in both episodes is summarised in Table 2.

**Table 2:** Fulfillment of HLH-2004 diagnostic criteria during both episodes. The table lists the eight diagnostic criteria defined by the hemophagocytic lymphohistiocytosis (HLH)-2004 protocol and indicates the presence or absence of each in the two episodes of HLH. A diagnosis requires at least five of these criteria.

Diagnostic criterion	Episode 1	Episode 2
Fever	Documented high-grade fever was present.	High-grade fever was present.
Splenomegaly	Splenomegaly was not reported in clinical records.	Splenomegaly was not reported.
Cytopenias	Pancytopenia involving all three cell lines was observed.	Cytopenias were present, affecting at least two hematologic lineages.
Hypertriglyceridemia and/or hypofibrinogenemia	These features could not be confirmed due to a lack of documented values in the available records.	These features could not be confirmed due to a lack of documented values in the available records.
Hemophagocytosis	Hemophagocytosis was confirmed on bone marrow aspirate and trephine biopsy (BMAT).	Hemophagocytosis was clinically presumed but not histologically confirmed.
Low or absent natural killer (NK) cell activity	NK cell activity was not tested during this episode.	NK cell function was not assessed.
Elevated serum ferritin (>500 ng/mL)	Serum ferritin was markedly elevated, consistent with HLH.	Serum ferritin was elevated beyond the diagnostic threshold.
Elevated soluble interleukin-2 receptor (sCD25)	Not assessed.	Not assessed.
≥5 of 8 HLH-2004 diagnostic criteria fulfilled	Yes – criteria were met based on clinical and laboratory data.	Yes – criteria were met despite some parameters being presumed or unavailable.

Given the autoimmune nature of this HLH flare-up, the patient was treated with anakinra. Corticosteroids were also used during the acute phase, and the patient was closely monitored with a plan for long-term follow-up.

**Figure 3:**
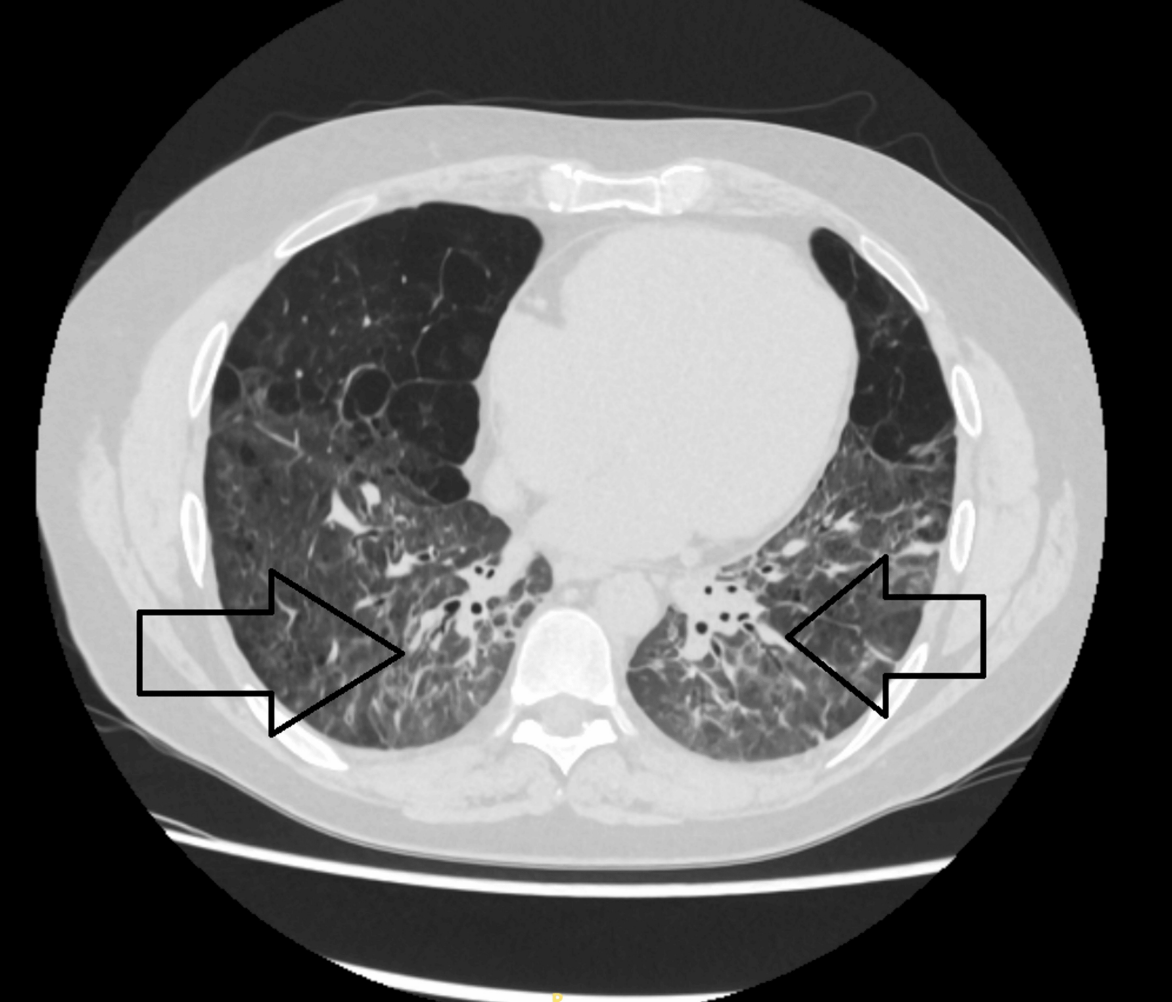
Axial CT chest (May 2025) shows near-complete resolution of lung infiltrates after anakinra therapy, with minimal residual fibrotic changes (black arrows). Improvements reflect IL-1 receptor antagonist treatment for MAS secondary to AOSD. CT: computed tomography; IL-1: interleukin-1; MAS: macrophage activation syndrome; AOSD: adult-onset Still’s disease

Both episodes of HLH were successfully managed with anakinra, and no further relapses have been reported at the time of writing. However, he remains under specialist respiratory follow-up due to the risk of HLH recurrence and potential chronic pulmonary complications. Near-complete radiological resolution after IL-1 receptor antagonist therapy is shown in Figure 3.

## Discussion

HLH is a rare but life-threatening hyperinflammatory syndrome characterized by excessive and dysregulated activation of macrophages, cytotoxic T lymphocytes, and NK cells. This immune overactivation results in uncontrolled cytokine release, leading to multiorgan dysfunction [1,2]. Although traditionally recognized in pediatric populations, adult-onset HLH is increasingly reported and poses significant diagnostic and therapeutic challenges due to variable triggers and nonspecific presentations that often mimic sepsis, malignancy, or autoimmune flares [3].

In the current case, the patient initially presented with gastrointestinal symptoms, pancytopenia, markedly elevated ferritin, and hemophagocytosis on bone marrow aspirate. These findings fulfilled the HLH-2004 diagnostic criteria and facilitated early recognition [1]. Parvovirus B19 and latent TB were identified as possible triggers during this initial episode. While infections remain the most common cause of secondary HLH, parvovirus B19-associated HLH is relatively rare, though confirmed in immunocompromised hosts by PCR detection [2,4]. The patient responded to dexamethasone and IVIG, with resolution of symptoms.

At recurrence, the trigger was AOSD, an autoinflammatory condition that can precipitate MAS, a recognized subtype of HLH. MAS-associated HLH is often relapsing and refractory to conventional regimens [3,5]. The novelty of this case lies in the recurrence of HLH in the same patient with two distinct etiologies, infectious (parvovirus B19/latent TB) and autoimmune (AOSD/MAS). Such dual-trigger recurrence is rarely described and underscores the heterogeneous mechanisms underlying HLH pathogenesis.

Importantly, in both episodes, the patient responded favorably to anakinra, an interleukin-1 receptor antagonist. Increasing evidence supports the use of cytokine-directed biologics such as anakinra, tocilizumab, and JAK inhibitors as effective and safer alternatives to cytotoxic agents in adult secondary HLH, particularly in MAS [5,6]. Retrospective studies and case series have shown rapid improvement, reduced mortality, and favorable safety profiles with intravenous anakinra in HLH/MAS [5,6,8]. This case reinforces the evolving role of biologic therapies in the management of recurrent or refractory adult HLH.

During the recurrent episode, the patient also developed pulmonary manifestations, with CT findings suggestive of organizing pneumonia and/or NSIP. Pulmonary involvement in HLH is underrecognized and may result from direct immune-mediated injury or secondary autoimmune inflammation [3,7]. Mechanistically, persistent cytokine excess can drive alveolar injury, immune-cell infiltration, and interstitial remodeling, contributing to chronic inflammatory lung disease in HLH/MAS. Organizing pneumonia has previously been reported in HLH triggered by chronic active EBV infection and other systemic inflammatory processes [7]. Distinguishing organizing pneumonia, NSIP, or other interstitial lung pathologies is clinically important for treatment planning and prognosis.

This case highlights several critical lessons. First, HLH should be considered in adults with unexplained cytopenias, hyperferritinemia, and systemic inflammation, particularly when there is multiorgan involvement. Second, recurrence may be driven by different triggers in the same patient, demanding careful surveillance and tailored therapy. Third, pulmonary complications may represent a long-term sequela of HLH recurrence and should prompt close radiologic and functional follow-up. Finally, cytokine-directed biologics such as anakinra offer a promising therapeutic avenue, particularly in MAS-associated HLH, and may provide durable control with fewer toxicities compared to cytotoxic regimens.

In summary, this case is notable for recurrent HLH triggered first by infection and later by autoimmunity, complicated by persistent pulmonary involvement, and successfully managed with targeted biologics. To our knowledge, few reports have described this combination, emphasizing the importance of individualized therapy and long-term multidisciplinary surveillance in HLH survivors.

## Conclusions

This case highlights the diagnostic and therapeutic challenges of HLH in adults, particularly when both infectious and autoimmune triggers are involved. The initial episode was likely triggered by parvovirus B19 and latent TB, while the recurrence was associated with AOSD, illustrating that HLH can recur with different etiologies. Successful treatment with anakinra, an interleukin-1 receptor antagonist, underscores the growing role of cytokine-targeted therapies as safer, effective alternatives in secondary HLH. Early recognition, prompt immunosuppressive therapy, and long-term follow-up are essential to prevent organ failure, monitor for relapse, and address complications such as pulmonary fibrosis. Clinicians should maintain a high index of suspicion for HLH in adults with systemic inflammation and multiorgan involvement and consider biologics when conventional therapies are inadequate or poorly tolerated.

## References

[1] Henter JI (2025). Hemophagocytic lymphohistiocytosis. N Engl J Med.

[2] George MR (2014). Hemophagocytic lymphohistiocytosis: review of etiologies and management. J Blood Med.

[3] Ponnatt TS, Lilley CM, Mirza KM (2022). Hemophagocytic Lymphohistiocytosis. Arch Pathol Lab Med.

[4] Pestana Santos C, Cruz D, Gonçalves de Sousa B, Judas T (2024). From diagnosis to treatment: a successful case of haemophagocytic lymphohistiocytosis of presumed bacterial aetiology in an adult. Eur J Case Rep Intern Med.

[5] Konkol S, Killeen RB, Rai M (2025). Hemophagocytic lymphohistiocytosis. StatPearls [Internet].

[6] Lee BJ (2024). Improved survival outcomes with anakinra over etoposide-based therapies for the management of adults with hemophagocytic lymphohistiocytosis: a retrospective multicenter research network study. Ther Adv Hematol.

[7] Wu X, Wang K, Gao Y (2021). Acute fibrinous and organizing pneumonia complicated with hemophagocytic lymphohistiocytosis caused by chronic active Epstein-Barr virus infection: a case report. BMC Infectious Diseases.

[8] Naymagon L (2022). Anakinra for the treatment of adult secondary HLH: a retrospective experience. Int J Hematol.

